# Frequency and Perception of Sexual Activity during Pregnancy in Iranian Couples

**Published:** 2012-06-19

**Authors:** Farahnaz Torkestani, Shahrzad Hadavand Hadavand, Zohreh Khodashenase, Sima Besharat, Ali Davati, Zohreh Karimi, Nafiseh Zafarghandi

**Keywords:** Sexual Intercourse, Pregnancy Outcome, Adverse Effect

## Abstract

**Background:**

Pregnancy stimulates partners to search for ways to preserve their mutual emotional relations and satisfy their sexual needs, with some limitations. This study evaluates the frequency and perception of sexual intercourse during pregnancy in a group of Iranian couples.

**Materials and Methods:**

In this cross-sectional study, 155 pregnant women were recruited from two academic clinics in Tehran. The exclusion criteria were: any underlying disease, history of pelvic surgery or gynecologic and obstetric complications, abortion or sterility, and previous preterm labor. A checklist was administrated in the labor room, that included: demographic data, partus and their viewpoints about sexuality. Frequency of sexual activity in each trimester, vaginal intercourse, coitus position, orgasm, breast stimulation, condom usage, and pregnancy outcome were recorded. Data were analyzed with t- and chi-square tests.

**Results:**

Women and their husbands with sexual behaviors during pregnancy had a lower mean age; the majority were nulipara (p<0.05). The biggest reason for decreased intercourse in the first trimester was fear of abortion (39.45%). No significant relationship between sexual activity in pregnancy and preterm labor, gestational age, membrane rupture, and fetal outcome was shown. There was a significant negative relationship between intercourse in the 2nd and 3rd trimesters and need to induction.

**Conclusion:**

Although our results showed that sexual intercourse had no adverse effect on the fetus and was a proper stimulus for the induction of delivery, its frequency was reduced during the gestational stage due to parents’ fear of adverse effects.

## Introduction

As shown by increasing evidence, sexual health issues significantly affect quality of life (1). Tremendous physical and psychological changes in the pregnancy period, along with cultural and social taboos, affect sexual behavior during pregnancy (2, 3).

A total of 86% to 100% of all couples are sexually active during pregnancy, but others usually report a decreased frequency of intercourse and sexual desire, particularly from the female (1). Pregnancy stimulates partners to search for ways to preserve their mutual emotional relations and sexual needs, with some limitations. Nausea and vomiting, hormonal changes, fear of adverse effects of intercourse (i.e., preterm labor) are among the most prevalent reasons for decreased libido, especially amongst women (4-6).

As pregnancy progresses, more restrictions on sexual function are imposed due to increased abdominal size, fear of harming the baby, and other factors that are mentioned in different studies (6).

Anxiety and inadequate knowledge, fear of bleeding or miscarriage, and infection of female genitalia are noted as the main concerns for avoiding sex during pregnancy (7-9).

Emotional distress caused by sexual dysfunction could also affect self-esteem and the relationship. It has been said that the sexual experience for a woman could be under the pressure of cultures or traditional context in the living environment of the pregnant woman and her husband (6).

This study was designed to evaluate the frequency and perception of sexual intercourse during pregnancy in a group of Iranian couples.

## Materials and Methods

This cross-sectional study was conducted in two academic clinics in Tehran, Iran during 2008. One hundred fifty five pregnant women were recruited by the simple sampling method. The exclusion criteria were as follows: any underlying disease (diabetes mellitus, hypertension, other systemic diseases), history of pelvic surgery (due to uterine anomaly, ovarian cysts, appendicitis, etc.), history of gynecologic and obstetric complications (bleeding during pregnancy, intra uterine growth retardation), previous history of abortion or sterility, and past history of preterm labor.

A checklist was administrated in the labor room that included demographic data, partus, and their views on sexuality during pregnancy. The frequency of sexual activity in each trimester, vaginal intercourse, coitus position, orgasm, breast stimulation, condom usage, gestational age, delivery method, need for induction, preterm labor (before 37 weeks), premature rupture of the membranes, APGAR, and fetal outcome were also recorded.

The Ethical Committee of Shahed University of Medical Sciences approved this study and informed consent was taken from all participants. Data were entered into SPSS version 16 software and analyzed with the t and chi-square tests.

## Results

We recruited 155 pregnant women to participate in the study. Of participants, 69 (44.5%) believed that intercourse was dangerous late in pregnancy, 44 (28.4%) thought it was dangerous during all trimesters, 18 (11.6%) said that their husbands believed it to be harmful, 16 (10.3%) believed it to be safe, and 8 (5.2%) had no information about their opinion.

In total, 124 women (80%) were sexually active during pregnancy. These couples had a lower mean age (p=0.02) than the non-sexually active couples (Table 1).

The frequency of intercourse was highest in the first trimester (36.8% had intercourse once a week), than in the second trimester (32.9% had intercourse once a month), and third trimester (49.7% reported no sexual intercourse; Table 2).

**Table 1:** Mean (± SD) of some variables between those with and without sexual activity during pregnancy


Sexual activity Variable	First trimester		Second trimester		Third trimester	

	No	Yes	No	Yes	No	Yes

**Mother age (Y)**	26.4 ± 4.2	26.4 ± 3.6	27 ± 3.8	25.84 ± 3.6	27.27 ± 4.3	26.2 ± 3.5
**BMI (Kg/m^2^)**	28.3 ± 4.4	29.6 ± 3.8	29.4 ± 4.08	29.2 ± 3.8	29.3 ± 4.01	29.3 ± 3.9
**Marriage duration (Y)**	4.7 ± 3.5	4.8 ± 3.7	4.6 ± 3.36	5.04 ± 3.9	5.2 ± 4.07	4.7 ± 3.5
**Father age (Y)**	31.6 ± 5.5	32.5 ± 4.8	30.6 ± 5.9	32.1 ± 4.8	31 ± 5	32.8 ± 6.6


**Table 2:** Frequency of intercourse in each trimester


Trimester	First	Second	Third
Frequency	No. (%)	No. (%)	No. (%)

**Every day**	1(0.6)	1 (0.6)	0
**Every other day**	7 (4.5)	2 (1.3)	1 (0.6)
**2 times a week**	18(11.6)	14(9)	7 (4.5)
**One in a week**	57(36.8)	43(27.7)	30(19.4)
**One in a month**	30(19.4)	51(32.9)	22(20.6)
**Occasionally**	11(7.1)	11(7.1)	8 (5.2)
**None**	31(20)	33(21.3)	87(56.13)
**Total**	155 (100)	155 (100)	155 (100)


The most prevalent etiology for the decreased times for intercourse in the first trimester was fear of abortion (39.45%), in the second trimester it was declining libido (26.28%), and in the third trimester it was fear of membrane rupture (19.3%).

No significant relationship between sexual activity during pregnancy and preterm labor, premature rupture of the membranes, and fetal outcome (birth weight, APGAR, need for NICU admission) was shown.

There was a significant negative relationship between intercourse in the second and third trimesters and the need for induction (p=0.03). The need for induction was reduced due to the increase in frequency of intercourse.

Vaginal intercourse, secretion of semen in the vagina, the position during intercourse (side-toside or man-on-top), breast stimulation, condom usage, and female orgasm were not related to preterm labor. These factors were not related to membrane rupture except for the presence of semen in the vagina during the third trimester (p=0.03).

## Discussion

According to the present study, the most prevalent reasons for the decreasing frequency of intercourse during pregnancy was fear of abortion (39.45%), declining libido in the second trimester (26.28%), and fear of membrane rupture in the third trimester (19.3%). No significant relationship was seen between sexual activity during pregnancy and preterm labor, gestational age, membrane rupture, and fetal outcome (birth weight, APGAR, need for NICU admission).

The rate of avoiding any kind increased during pregnancy (20% in the first trimester, 21.3% in the second, and 49.7% in the third). This pattern has also been reported in other studies.

Leite et al. conducted a cohort study on 271 healthy Brazilian pregnant women. The rate of sexual dysfunction significantly decreased during the pregnancy as follows: 46.6% in the first, 34.2% in the second, and 73.3% in the third trimester (1). A similar result was reported among Thai (10), Chinese (7), Polish (8), and Turkish pregnant women (11).

In the present study, there was a significant relationship between intercourse in the second and third trimesters and the need to induce labor. But in a study in Kuala Lumpur, the results of 210 pregnant women showed no differences between the groups who had coitus before delivery and the control group in regards to the rate of spontaneous labor (1). In Thailand, women with infrequent sexual intercourse early in pregnancy had a lower incidence of recurrent spontaneous preterm labor (28% vs. 38%) (10).

## Conclusion

This study suggests that sexual intercourse does not adversely affect the fetus; it is a proper stimulus for the induction of delivery. The low rate of sexual activity in our study, regardless of the trimesters of the pregnancy, raises an important question about the taboo of sexual intercourse during pregnancy. It could be related to a cultural background in which women avoid speaking about their desires and sexual needs more attention should be pay.

It has been discussed in other studies that the low interest and insufficient knowledge of health care providers on the issue of sexuality during pregnancy can lead to a lower amount information given to patients, and this is among the most common reason for the lack of discussion on this topic (12).

## References

[1] Leite AP, Campos AA, Dias AR, Amed AM, De Souza E, Camano L (2009). Prevalence of sexual dysfunction during pregnancy. Rev Assoc Med Bras.

[2] Bartellas E, Crane JM, Daley M, Bennett KA, Hutchens D (2000). Sexuality and sexual activity in pregnancy. BJOG.

[3] Shojaa M, Jouybari L, Sanagoo A (2009). The sexual activity during pregnancy among a group of Iranian women. Arch Gynecol Obstet.

[4] Malarewicz A, Szymkiewicz J, Rogala J (2006). Sexuality of pregnant women. Ginekol Pol.

[5] Senkumwong N, Chaovisitsaree S, Rugpao S, Chandrawongse W, Yanunto S (2006). The changes of sexuality in Thai women during pregnancy. J Med Assoc Thai.

[6] Bello FA, Olayemi O, Aimakhu CO, Adekunle AO (2011). Effect of pregnancy and childbirth on sexuality of women in ibadan, Nigeria. ISRN Obstet Gynecol.

[7] Fok WY, Chan LY, Yuen PM (2005). Sexual behavior and activity in Chinese pregnant women. Acta Obstet Gynecol Scand.

[8] Sipiñski A, Kazimierczak M, Buchacz P, Sipiñska K (2004). Sexual behaviors of pregnant women. Wiad Lek.

[9] Dwyer JM (2001). High-risk sexual behaviours and genital infections during pregnancy. Int Nurs Rev.

[10] Yost NP, Owen J, Berghella V, Thom E, Swain M, Dildy GA 3rd (2006). Effect of coitus on recurrent preterm birth. Obstet Gynecol.

[11] Gökyildiz S, Beji NK (2005). The effects of pregnancy on sexual life. J Sex Marital Ther.

[12] Brtnicka H, Weiss P, Zverina J (2009). Human sexuality during pregnancy and the postpartum period. Bratisl Lek Listy.

